# Adjunct EF-M2 therapy improves clinical activity, steroid-sparing, and macrophage-linked biomarkers in feline chronic enteropathy: A randomized, double-blind, and placebo-controlled trial

**DOI:** 10.14202/vetworld.2025.3914-3928

**Published:** 2025-07-14

**Authors:** Evgeny Pokushalov, Claire Garcia, John Smith, Dmitry Kudlay, Nikolai Revkov, Anastasya Shcherbakova, Michael Johnson, Richard Miller

**Affiliations:** 1 Scientific Research Laboratory, Triangel Scientific, San Francisco, CA 94101, USA; 2 Department of Science, Center for New Medical Technologies, 630090 Novosibirsk, Russia; 3 Department of Pharmacy, Institute of Pharmacy, I.M. Sechenov First Moscow State Medical University (Sechenov University), 119435 Moscow, Russia; 4 Department of Pharmacy, Novosibirsk State University, 630090 Novosibirsk, Russia; 5 VEGA Veterinary Clinic, 630049 Novosibirsk, Russia; 6 BALTO Veterinary Clinic, 630055 Novosibirsk, Russia

**Keywords:** CLEC10A, EF-M2, feline chronic enteropathy, M2 polarization, macrophage-programming, spec fPL, steroid-sparing, triaditis

## Abstract

**Background and Aim:**

Feline chronic enteropathy (CE), often manifesting along the triaditis-axis with concurrent pancreatitis, remains difficult to manage despite standardized dietary modification and cobalamin supplementation. Dysregulated macrophage activity contributes to persistent mucosal and pancreatic inflammation. EF-M2 (Immutalon^™^, Activator MAF LLC, Russia) is an analytically defined, alpha-N-acetylgalactosamine (α-GalNAc) –bearing Gc protein-derived macrophage-activating factor 2.0 (GcMAF 2.0) ligand designed to engage C-type lectin domain family 10 member A (CLEC10A) and promote M2-leaning macrophage-programming. This study aimed to evaluate whether adjunct EF-M2 improves clinical disease activity compared with placebo and to determine whether clinical responses align with macrophage-linked pharmacodynamic (PD) biomarkers.

**Materials and Methods:**

A multicenter, randomized, double-blind, placebo-controlled, parallel-group trial was conducted in client-owned cats with CE (modified intention-to-treat = 36). Cats received EF-M2 or volume-matched saline twice weekly for 4 weeks in addition to standardized diet/B12 care, followed by a 4-week off-drug period (day 56). The primary endpoint was the change in the feline CE activity index (FCEAI) at day 28. Secondary outcomes included responder rate (≥50% reduction), steroid-sparing effect, serum specific feline pancreatic lipase (Spec fPL), blinded abdominal ultrasonography, PD markers arginase-1 to inducible nitric oxide synthase (ARG1/iNOS) ratio, interleukin-10 [IL-10], and tumor necrosis factor-alpha [TNF-α]). Safety was assessed using Veterinary Cooperative Oncology Group – Common Terminology Criteria for Adverse Events (VCOG-CTCAE) criteria.

**Results:**

EF-M2 significantly improved FCEAI scores at day 28 compared with placebo (least-squares mean difference −2.5; 95% confidence interval −3.7 to −1.3; p = 0.0007). Responder rates were higher with EF-M2 (61% vs. 28%), and more cats remained steroid-free through day 28 (72% vs. 39%). Clinical benefits partially persisted to day 56 (between-group difference in FCEAI −2.1; p = 0.004). In the pancreatitis-positive subgroup, EF-M2 produced a greater reduction in Spec fPL (−2.1 vs. −0.3 µg/L; p = 0.009) and improved pancreatic ultrasonography indices. PD markers shifted consistently with the intended mechanism (ARG1/iNOS ↑, IL-10 ↑, TNF-α ↓; all p ≤ 0.01), and ΔARG1/iNOS correlated with ΔFCEAI (r = −0.57; p = 0.001). Adverse events were mild and comparable between groups, with no treatment-related serious events.

**Conclusion:**

Short-course adjunct EF-M2 achieved clinically meaningful improvement in disease activity, reduced steroid exposure, and improved pancreatitis-associated indicators in cats with CE. The coherent M2-leaning PD signature supports macrophage-programming as a biologically plausible mechanism. EF-M2 demonstrated favorable tolerability and represents a promising adjunctive option for triaditis-axis disease.

## INTRODUCTION

Feline “triaditis–”, the concurrent occurrence of chronic enteropathy (CE), pancreatitis, and, in many cases, cholangitis, remains a common and challenging syndrome in referral practice [1]. Clinical signs are nonspecific, fluctuate over time, and overlap among the involved organs, making diagnosis and monitoring difficult [1, 2]. Current management focuses on dietary modification using hydrolyzed or novel protein diets, cobalamin supplementation when indicated, supportive control of nausea and pain, and glucocorticoid therapy [2, 3]. However, many cats achieve only partial symptomatic control and accumulate risks associated with prolonged steroid exposure [4]. These limitations highlight the need for adjunctive therapies with well-defined mechanisms that target upstream drivers of mucosal and pancreatic inflammation while preserving antimicrobial defense.

Macrophages play a central role in maintaining epithelial barrier integrity and orchestrating the resolution of inflammation [5–7]. Their functional states span a continuum, commonly described as pro-inflammatory “M1-like” and proresolving “M2-like” phenotypes. M2-skewed states, marked by elevated interleukin-10 (IL-10) and arginase-1 activity, are associated with tissue protection and repair in intestinal models, whereas persistent M1 polarization promotes epithelial injury, pain, and chronic inflammation [5–9]. Rather than relying on broad immunosuppression, recalibrating myeloid cell behavior through targeted “programmable” macrophage modulation has therefore emerged as a promising therapeutic concept [5, 7].

One strategically tractable pathway involves the C-type lectin receptor CLEC10A (macrophage galactose-type lectin, CD301), which recognizes terminal N-acetylgalactosamine (GalNAc) residues and is expressed on macrophage and dendritic cell subsets [10, 11]. Ligand engagement induces receptor internalization and signaling that can shift myeloid responses toward IL-10–dominant, pro-resolution outputs [10, 12]. This suggests that precise CLEC10A activation may function as a biological “dimmer switch,” tuning macrophage programs toward resolution without suppressing essential antimicrobial functions.

Vitamin D–binding protein–derived glycoproteins, historically grouped as GcMAF, display terminal N-acetylgalactosamine (GalNAc) motifs capable of engaging CLEC10A [10, 11, 13]. However, early GcMAF literature is confounded by retractions, unregulated production, batch heterogeneity, and endotoxin contamination, an important concern because picogram-level endotoxin can artifactually activate myeloid cells [14–18]. Modern development, therefore, requires rigorous analytical characterization, including glycoidentity, purity, potency, and adherence to international endotoxin limits (e.g., International Council for Harmonisation guideline Q6B (ICH Q6B); United States Pharmacopeia/European Pharmacopoeia (USP/Ph. Eur.) [17, 18].

Within this contemporary framework, we evaluated EF-M2 (Immutalon^™^, Activator MAF LLC, Russia), an analytically defined, α-GalNAc–bearing Vitamin D–binding protein derivative engineered to bias macrophages toward an M2-like, IL-10–dominant phenotype [10, 11, 13, 17, 18]. Designed to overcome historical shortcomings of earlier GcMAF preparations, EFM2 adheres to strict specifications for mono–α-GalNAc glycoidentity, high purity, low endotoxin content, and verified CLEC10A engagement. We conducted a randomized, double-blind, placebo-controlled add-on trial in cats with CE, with or without chronic pancreatitis, using the feline CE activity index (FCEAI) as the primary endpoint [19]. PD markers (ARG1/iNOS ratio, IL-10, tumor necrosis factor-alpha [TNF-α]) were embedded to determine whether clinical outcomes aligned with the predicted M2-shift signature.

Despite advances in diagnostic imaging, dietary management, and supportive care, the therapeutic landscape for feline CE, particularly when complicated by pancreatitis along the triaditis-axis, remains limited. Current treatment strategies rely heavily on glucocorticoids, which may induce only partial remission, exhibit variable long-term efficacy, and carry well-documented risks, including diabetes mellitus and immunosuppression. Importantly, no approved or widely adopted adjunctive therapies directly target the underlying immunopathology driving intestinal–pancreatic inflammation. Although macrophage dysregulation is increasingly recognized as a central mechanism in CE, there is a complete absence of controlled clinical trials evaluating biologics that modulate macrophage phenotype in cats. Furthermore, past interest in GcMAF-like agents has been undermined by nonstandardized manufacturing, unverified molecular identity, and confounding endotoxin contamination, leaving a scientific and clinical void regarding receptor-specific macrophage-programming in companion animals. As a result, the field lacks rigorously conducted, analytically validated, blinded trials that test whether targeted myeloid modulation can translate into measurable clinical benefit, steroid-sparing, and improvement in pancreatic biomarkers within the triaditis spectrum. This gap is particularly important given the need for disease-modifying rather than purely symptomatic therapies.

This study aimed to generate controlled, mechanistic, and clinically relevant evidence on a next-generation macrophage-programming biologic for feline CE. Specifically, we sought to determine whether adjunctive EF-M2, an analytically defined, mono–α-GalNAc, CLEC10A-targeted ligand designed to promote an M2-leaning macrophage phenotype, can enhance clinical outcomes when added to standardized diet/B12 care in cats with CE, with or without chronic pancreatitis. The primary objective was to evaluate its effect on disease activity using the FCEAI under a randomized, double-blind, and placebo-controlled framework. Secondary aims were to assess steroid-sparing potential, improvement in pancreatic biomarkers (Spec fPL and ultrasonography), and coherence between clinical outcomes and predefined PD signatures (ARG1/iNOS ratio, IL-10, TNF-α). Finally, we aimed to establish whether EF-M2 can deliver clinical benefit with a favorable safety profile, thereby providing the first evidence-based rationale for macrophage-targeted immunomodulation in feline CE and triaditis-axis disease.

## MATERIALS AND METHODS

### Ethical approval

The study protocol FELINE-TRIAD-1 (version 1.0; January 12, 2025) was reviewed and approved by the Institutional Animal Care and Use Committees of both participating companion-animal clinics: Veterinary Clinic A (protocol VCA2025017; approval date January 28, 2025) and Veterinary Clinic B (protocol VCB2025024; approval date January 29, 2025).

All procedures involving animals were conducted in accordance with the respective institutional guidelines for the care and use of animals in research, national regulations governing the use of client-owned companion animals for scientific purposes, and the Animal Research: Reporting of *In Vivo* Experiments 2.0 guidelines.

Only client-owned cats presented for investigation and management of naturally occurring CE, with or without chronic pancreatitis, were considered for enrolment. No experimental disease induction or non-clinical housing was performed. Before any study-specific procedures, owners received written and verbal information about the trial objectives, potential benefits and risks, permitted rescue medications, and their right to withdraw at any time without prejudice to ongoing clinical care. Written informed consent was obtained from all owners, including permission to use anonymized clinical data and ultrasound images for research and publication.

Animals remained under the care of their primary attending clinicians at each site and were managed according to best-practice standards for feline gastroenterology. Sedation, analgesia, antiemetic therapy, appetite stimulants, and other supportive measures were provided as clinically indicated. The number of cats enrolled was prospectively justified using a priori power calculations to minimize unnecessary exposure while retaining adequate statistical power. Safety was monitored throughout using the Veterinary Cooperative Oncology Group – Common Terminology Criteria for Adverse Events criteria, with predefined rules for rescue prednisolone, treatment interruption, or withdrawal in the event of clinically relevant deterioration. An independent data and safety monitoring process oversaw the accumulating safety data to ensure that no cat remained on-study treatment in the presence of unacceptable adverse effects.

### Study period and location

The study was conducted between February 2025 and August 2025 at two referral companion-animal clinics in Novosibirsk, Russian Federation: VEGA Veterinary Clinic and BALTO Veterinary Clinic. All enrolled animals were client-owned cats with naturally occurring CE, with or without chronic pancreatitis.

### Study design and oversight

This was a multicenter, randomized, double-blind, placebo-controlled, parallel-group clinical trial evaluating the efficacy and safety of Immutalon as add-on therapy to standard of care (SoC) in client-owned cats with CE (inflammatory bowel disease) with or without chronic pancreatitis. The active treatment period lasted for 4 weeks, followed by a 4-week off-drug observation (day 56). The trial was conducted at two veterinary clinics for companion animals. All study operations (design finalization, conduct, monitoring, data management, and statistical analysis) were performed independently by investigators from the Center for New Medical Technologies (Novosibirsk, Russia) and the Triangel Scientific Research Laboratory (San Francisco, USA).

### Determination of sample size

A sample of ~15 cats per arm was estimated to provide 80% power (two-sided α = 0.05) to detect an additional 2.5-point reduction in FCEAI at day 28 versus placebo (assumed standard deviation [SD] ≈2.3) using a two-sample t-test. To allow for ≈15% attrition, randomization was planned for 36 cats (18/arm). Recognizing potential undercoverage for smaller effects (δ≈2.0), SAP specified the use of repeated-measures models and hierarchical testing to improve efficiency and control multiplicity.

The sample size computations were performed using SAS PROC POWER, version 9.4 (SAS Institute Inc., Cary, NC, USA) and were cross-checked in R (R Foundation for Statistical Computing, Vienna, Austria; “pwr” package, version 1.3). Assumptions were: Two-sided α = 0.05, power = 0.80, between-group δ = 2.5 points on FCEAI with SD ≈ 2.3 (standardized effect ≈ 1.1). The δ assumption was anchored to (i) internal prescreening data under standardized SoC, indicating ≈2 2-point mean improvement by day 28, and (ii) the intent to detect an add-on effect exceeding that background variance. Allowing for ≈15% attrition, n = 18/arm was targeted. The primary analysis of covariance (ANCOVA) (with baseline FCEAI as a covariate) was expected to reduce residual variance (ρ ≈ 0.45, from baseline to change), providing additional efficiency beyond the t-test approximation used for planning.

### Animals

**Figure 1:**
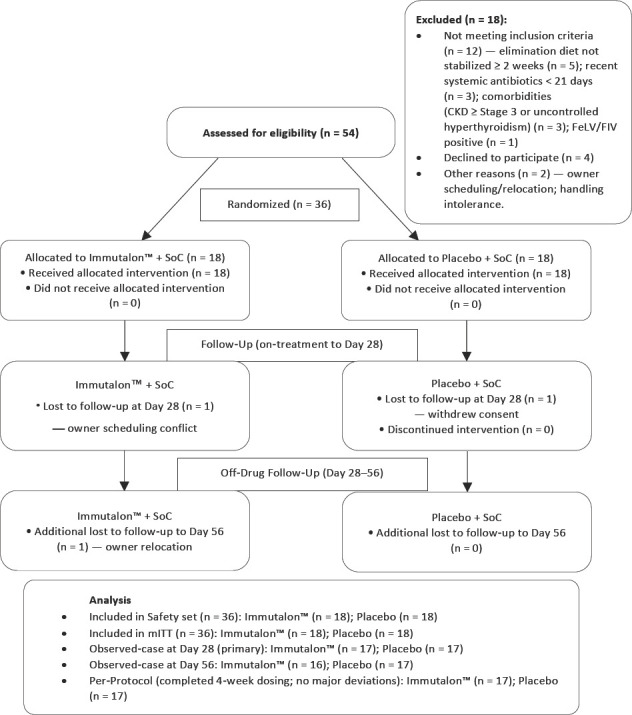
CONSORTVet flow diagram of cats enrolled in FELINETRIAD1 (randomized, double-blind, placebo-controlled add-on trial in feline chronic enteropathy/triaditis).

Client-owned cats aged ≥1 year and weighing 2–10 kg with stable clinical status were eligible. The key inclusion criteria were as follows: (i) Chronic gastrointestinal signs for ≥3 weeks; (ii) FCEAI between 5 and 12 at baseline; (iii) objective confirmation of CE by ultrasound features and, where available, endoscopy/biopsy in a predefined subcohort (target ≥12); and (iv) evidence of pancreatic involvement defined by serum Spec fPL >5.3 μg/L and/or ultrasonographic features of chronic pancreatitis. Feline leukemia virus/feline immunodeficiency virus (FeLV/FIV) tests were negative in all cats; cobalamin deficiency, if present, was corrected before randomization; and helminth control was verified by negative flotation or a short deworming course before enrolment. The exclusion criteria were as follows: Severe systemic illness; chronic kidney disease International Renal Interest Society stage ≥3; decompensated hyperthyroidism; inoperable neoplasia; infectious enteritis (e.g., parvovirus/panleukopenia); use of systemic immunosuppressants other than rescue prednisolone within 30 days; use of systemic antibiotics within 21 days unless a confirmed bacterial indication was present; marked cachexia; and pregnancy or lactation (Figure 1).

### Randomization, allocation concealment, and masking

Cats were randomly assigned (1:1) to Immutalon + SoC or placebo + SoC using computer-generated block randomization (block size = 4) stratified by site and pancreatitis status (yes/no). Owners, attending clinicians, ultrasonographers, and laboratory personnel were blinded throughout the study. Injection volumes, syringes, and schedules were identical between groups; when the protocol allowed an intensified dosing interval (every 48 h) for severe cases, blinding was preserved with mirrored placebo injections. Owners and attending clinicians completed a blinding questionnaire (“active,” “placebo,” or “unsure”) on day 56. Bang’s blinding index (BBI) was computed with a 95% confidence interval (CI). Owner BBI was 0.03 (95% CI −0.22 to +0.27) and clinician BBI −0.05 (95% CI −0.29 to +0.18), indicating effective masking; correct guess rates did not differ from chance (both p > 0.40).

### Interventions

Immutalon (EF-M2; glyco-engineered GcMAF analogue) was administered subcutaneously at 1 μg in 1 mL twice weekly (approximately every 72 h) for 4 weeks to cats ≤12 kg; for heavier animals, dosing was 0.1 μg/kg. In cases with severe disease or mixed infections, interval reduction to every 48 h was permissible under monitoring committee oversight; to maintain masking, an identical mirrored schedule was used in the placebo arm. The placebo consisted of 0.9% sodium chloride at an identical volume and frequency.

### Analytical definition of EF-M2

#### Identity and glycoform

LC-MS/MS glycopeptide mapping (tryptic digest, Byonic-assisted) demonstrated a single oglycan with terminal α GalNAc on the vitamin D–binding protein domain III (Thr420) without additional sialylation or elongation. Orthogonal confirmation was performed using Vicia villosa agglutinin (VVA) lectin blot (α GalNAc specific) and exoglycosidase trimming (α N acetylgalactosaminidase abolishing VVA reactivity), consistent with mono α GalNAc display.

#### Molecular mass and purity

The intact mass electrospray ionization time-of-flight mass spectrometry yielded 53,618 ± 12 Da. Sodium dodecyl sulfate–polyacrylamide gel electrophoresis (SDS-PAGE) densitometry indicated 96.2% ± 0.7% purity of the main band; reversed-phase high-performance liquid chromatography (RP-HPLC) area purity was 97.8% ± 0.6%. Size-exclusion HPLC showed aggregates ≤0.5% and fragments ≤1.5% of the total area.

#### Endotoxin and bioburden control

Endotoxin was quantified using a kinetic chromogenic limulus amebocyte lysate assay. The lot release criterion was ≤0.05 endotoxin units (EU)/µg protein; clinical lots used in this trial tested 0.007, 0.011, and 0.004 EU/µg. The material was 0.22 µm sterile-filtered; sterility testing returned negative results before release.

#### CLEC10A receptor-engagement

Binding to recombinant human and feline CLEC10AFc was assessed at 25°C by biolayer interferometry. The apparent equilibrium dissociation constants were K_D (hCLEC10A) = 0.85 µM (k_on = 7.2 × 104 M¹/s; k_off = 0.061 s¹) and K_D (fCLEC10A) = 1.1 µM. No specific binding was observed to negative control lectins lacking terminal GalNAc specificity. Assay performance was verified by competition with free GalNAc (IC_50_ = 2.8 mM), supporting α GalNAc-dependent attachment. In contrast to earlier “GcMAF” preparations, EFM2 lots are specified by defined glycoidentity, purity, low endotoxin release limits, and documented CLEC10A affinity, minimizing the risk of myeloid readouts due to lipopolysaccharide contamination or batch heterogeneity.

### Standard care and rescue therapy

All cats received a standardized SoC protocol: (i) An elimination diet (hydrolyzed or novel protein) instituted ≥2 weeks before randomization and maintained unchanged; (ii) parenteral cobalamin supplementation for documented deficiency; and (iii) permitted symptomatic therapies (e.g., maropitant or ondansetron for nausea, mirtazapine for appetite stimulation, and buprenorphine for pain). Rescue prednisolone (1 mg/kg once daily with taper) was allowed according to a predefined algorithm if by day 14 the FCEAI had not improved by ≥1.5 points, or if ≥3 vomiting episodes/day occurred, or if body weight loss was documented to be >5%. The cumulative prednisolone dose was analyzed as a secondary outcome.

### Procedures and assessments

Study timeline: Screening (day −14 to 0) → day 0 (randomization) → days 7, 14, 21, and 28 (end of dosing) → day 56 (off-drug follow-up). At each on-study visit, the following were collected: FCEAI; owner diaries of vomiting and stool; body weight and body condition score; owner-reported quality of life (0–10 Likert scale; higher scores indicating better status); adverse events (AE); serum biochemistry (alanine transaminase [ALT], alkaline phosphatase, gamma-glutamyl transferase (GGT), bilirubin, creatinine); and complete blood count. The owner’s diaries captured daily vomiting episodes and stool consistency. Compliance was defined as the number of days with both fields recorded during each 7-day window. Across 144 planned weekly windows (36 cats × 4 weeks), 135 (94%) were complete (≥5/7 days recorded), 7 (5%) were partial (3–4/7 days; missing days imputed by the within cat mean of recorded days within that window), and 2 (1%) were missing and treated as missing-at-random in the multiple-imputation (MI) framework. The MI framework was used to assess the number of missing days. Window-level compliance did not differ between arms (Immutalon 95% vs. placebo 93%, χ^2^ p = 0.62). Diary entries were time-stamped and cross-checked against FCEAI component visits; biologically implausible values (e.g., >10 vomiting episodes/day) triggered source verification before lock. Spec fPL was measured on days 0, 28, and 56, and Vitamin B12/folate was measured on days 0 and 28. Blinded abdominal ultrasound was performed on days 0, 28, and 56 to derive a composite index for small intestinal mucosal thickness and layering and pancreatic echogenicity. PD biomarkers were assayed in serum at baseline, day 14, and day 28: ARG1/iNOS ratio, IL-10, and TNF-α. The exploratory endpoints included fecal calprotectin/α1-proteinase inhibitor, 16S microbiome profiling in a subcohort (target n ≈ 15), and antidrug antibodies (ADA).

### Outcomes

The primary endpoint was the change in FCEAI from baseline to day 28. Key secondary endpoints (tested hierarchically) were as follows: (1) Responder proportion at day 28 (≥50% reduction in FCEAI; remission defined as FCEAI ≤3 was also captured and summarized at days 28/56); (2) cumulative prednisolone dose to day 28 and the proportion of steroid-free cats; (3) change in Spec fPL at day 28; (4) change in body weight; (5) change in the ultrasound composite index; and (6) change in PD markers (ARG1/iNOS ratio, IL-10, TNF-α) to day 28. Safety endpoints included the incidence and severity of treatment-emergent adverse events (TEAE), graded according to Veterinary Cooperative Oncology Group – Common Terminology Criteria for Adverse Events version 2.1 (VCOG-CTCAE v2.1), laboratory abnormalities, and local injection site reactions.

### Statistical analysis

Beyond the primary ANCOVA, longitudinal endpoints were analyzed using a mixed model for repeated-measures (MMRM) with unstructured covariance (Kenward–Roger degrees of freedom), and missing values were imputed with concordant sensitivity analyses under a joint-model missing at random (MAR) assumption. We applied a fixed-sequence hierarchical gatekeeping procedure aligned with human randomized controlled trial (RCT) practice to control family-wise type I error across key secondaries (gate order: Responder rate → steroid-sparing [steroid-free proportion; cumulative prednisolone] → Spec fPL → ultrasound composite index → body weight → PD suite [ARG1/iNOS, IL-10, TNF-α]). All analyses followed the pre-finalized SAP and were executed blinded to allocation. Extreme responses were predefined as ΔFCEAI ≤−8 (marked improvement) or ≥+3 (paradoxical worsening) from baseline to day 28, and no data points were removed from the primary analyses. Robust sensitivity evaluations included (i) refitting the primary ANCOVA after winsorization at the 2.5^th^/97.5^th^ percentiles, (ii) leave-one-out influence tests, and (iii) exclusion of predefined extremes; these were *post hoc*, nominal, and descriptively reported. Because an externally validated minimal clinically important difference (MCID) for FCEAI is not available, we contextualized effect magnitude using distribution-based effect sizes (Hedges g) derived from the ANCOVA model and a number needed to treat (NNT) for the ≥50% responder definition, calculated from the observed control risk. The efficacy analyses followed the modified intention-to-treat (mITT) principle (all randomized cats with ≥1 post-baseline FCEAI), while a per-protocol (PP) set excluded major protocol deviations, and the safety set included all cats receiving ≥1 dose. The primary analysis used ANCOVA with treatment arm, center, and pancreatitis status as fixed effects and baseline FCEAI as a covariate (two-sided α = 0.05). Longitudinal FCEAI was additionally analyzed using an MMRM with an unstructured covariance matrix across days 7/14/21/28. The Cochran–Armitage trend test, with logistic models reporting odds ratios (OR) and 95% CIs, was used for responder analyses. Prespecified PD–clinical correlations employed Pearson or Spearman coefficients for ΔFCEAI versus ΔARG1/iNOS and ΔIL-10/ΔTNF-α. Multiplicity across secondary endpoints was controlled through hierarchical testing, and the remaining endpoints were descriptively summarized. Missing data were addressed using multiple imputation under a joint-model missing-at-random assumption, with last observation carried forward and PP analyses used for sensitivity assessments.

An independent monitor verified the source data at 100% for PD biomarkers. A Data and Safety Monitoring Board reviewed accumulating safety data at 2-week intervals, and prespecified safety stopping rules were in place. Allocation concealment and blinding procedures were enforced at sites; all imaging and laboratory assessments were performed by blinded personnel.

## RESULTS

### Animal disposition and baseline characteristics

A total of 54 cats were screened, of which 36 were randomized (n = 18 per arm) and included in both the mITT and safety sets. Of these, 34 completed the day 28 visit, and 33 completed the day 56 follow-up. No cat discontinued the study due to AEs (Figure 1). Baseline characteristics were well balanced between treatment groups owing to stratification by site and pancreatitis status. The mean baseline FCEAI was 7.4 ± 1.6 in both groups (standardized mean difference [SMD] = 0.00), and other demographics, disease history, laboratory values, and ultrasound indices were similarly comparable (Table 1). All cats were FeLV/FIV-negative; elimination diets were stabilized for ≥2 weeks before randomization, and any cobalamin deficiency was corrected before day 0.

**Table 1:** Baseline characteristics of the randomized population (mITT). Data are mean ± SD or n/n (%) unless otherwise specified; for skewed variables, median (IQR).

Domain or characteristic	Immutalon + SoC (n = 18)	Placebo + SoC (n = 18)	Total (n = 36)	SMD (key)
Randomization strata				
Site 1/site 2, n	9/9	9/9	18/18	—
Pancreatitis status (yes), n (%)	10 (56)	10 (56)	20 (56)	—
Demographics				
Age, years	8.7 ± 3.6	8.9 ± 3.4	8.8 ± 3.5	0.06
Age categories, n (%) - ≤5/6–10/≥11	4 (22)/9 (50)/5 (28)	5 (28)/8 (44)/5 (28)	9 (25)/17 (47) /10 (28)	—
Sex, n (%) - male/female	10 (56)/8 (44)	9 (50)/9 (50)	19 (53)/17 (47)	—
Neutered/spayed, n (%)	17 (94)	16 (89)	33 (92)	—
Breed, n (%) - Domestic shorthair/domestic longhair/Purebred	13 (72)/3 (17)/2 (11)	12 (67)/3 (17)/3 (17)	25 (69)/6 (17)/5 (14)	—
Anthropometrics and general status				
Body weight (kg)	4.4 ± 0.9	4.5 ± 0.8	4.5 ± 0.8	0.12
Body condition score (1–9)	4.8 ± 1.0	4.9 ± 0.9	4.8 ± 1.0	—
Disease history and clinical signs				
Duration of GI signs, months, and median (IQR)	6.0 (4.0–9.0)	6.0 (3.5–8.5)	6.0 (4.0–9.0)	—
Vomiting episodes per 7 days	3.1 ± 1.6	3.0 ± 1.5	3.1 ± 1.5	0.06
Stool score (0–3, higher = better)	1.2 ± 0.5	1.3 ± 0.5	1.2 ± 0.5	0.20
Owner-reported appetite (0–10; higher = better)	4.8 ± 1.5	4.9 ± 1.4	4.8 ± 1.5	—
Owner-reported quality of life (0–10)	5.2 ± 1.6	5.1 ± 1.5	5.1 ± 1.6	0.06
Abdominal pain on palpation, n (%)	8 (44)	7 (39)	15 (42)	—
Indices and markers of pancreatitis				
FCEAI, baseline	7.4 ± 1.6	7.4 ± 1.6	7.4 ± 1.6	0.00
Spec fPL, μg/L	6.8 ± 2.1	6.9 ± 2.2	6.9 ± 2.1	0.05
Laboratory profile				
Cobalamin deficiency at screening, n (%)	7 (39)	6 (33)	13 (36)	—
Serum cobalamin level at baseline (post correction), pmol/L	387 ± 112	379 ± 120	383 ± 116	—
Serum folate, ng/mL	12.1 ± 3.8	11.8 ± 4.2	11.9 ± 4.0	—
ALT, U/L, and median (IQR)	68 (52–92)	70 (50–95)	69 (51–93)	—
ALP, U/L, and median (IQR)	39 (28–58)	41 (29–62)	40 (28–60)	—
GGT, U/L, and median (IQR)	3 (2–6)	3 (2–5)	3 (2–6)	—
Total bilirubin, mg/dL, median IQR	0.2 (0.1–0.3)	0.2 (0.1–0.3)	0.2 (0.1–0.3)	—
Creatinine, mg/dL	1.2 ± 0.3	1.1 ± 0.3	1.1 ± 0.3	—
WBC, 10^9^×/L	8.3 ± 2.2	8.1 ± 2.0	8.2 ± 2.1	—
Ultrasound (blinded protocol)				
Composite small-intestinal + pancreatic US index (sum score)	6.5 ± 2.0	6.3 ± 2.1	6.4 ± 2.0	0.10
Pancreatic echogenicity, grade 0–3, median interquartile range	2 (1–3)	2 (1–3)	2 (1–3)	—
Hepatobiliary involvement on US, n (%)	6 (33)	5 (28)	11 (31)	—
Diagnostics and prior/concomitant care				
Endoscopy/biopsy performed at baseline, n (%)	6 (33)	6 (33)	12 (33)	—
FeLV/FIV-negative, n (%)	18 (100)	18 (100)	36 (100)	—
Systemic antibiotics in the previous 21 days, n (%)	1 (6)	2 (11)	3 (8)	—
Systemic immunosuppressants in the previous 30 days, n (%)	0 (0)	0 (0)	0 (0)	—
Concomitant symptomatic therapy at baseline, n (%)	7 (39)	8 (44)	15 (42)	—

SMD = Standardized mean difference, IQR = Interquartile range, SD = Standard deviation, FCEAI = Feline chronic enteropathy activity index, WBC = White blood cells, ALT = Alanine transaminase, ALP = Alkaline phosphatase, GI = Gastrointestinal, Spec fPL = Specific feline pancreatic lipase, GGT = Gamma-glutamyl transferase, FeLV/FIV = Feline leukemia virus/feline immunodeficiency virus.

In the biopsy subcohort (n = 12), central histopathology adjudication using the American College of Veterinary Internal Medicine/World Small Animal Veterinary Association (ACVIM/WSAVA) framework showed 7/12 mild lymphoplasmacytic enteritis, 3/12 moderate lymphoplasmacytic enteritis, and 2/12 eosinophilic enteritis. Median baseline grades were: Villus atrophy 1 (0–2), lamina propria lymphoplasmacytic infiltration 2 (1–2), and crypt architectural change 0 (0–1). Post-treatment biopsies were not mandated for ethical and pragmatic reasons.

### Primary efficacy outcome

By day 28, the LS-mean reduction in FCEAI was greater in the Immutalon + SoC group (−4.8 points) than in the placebo + SoC group (−2.3 points), resulting in a between-group difference of −2.5 points (95% CI, −3.7 to −1.3; p = 0.0007). Although no externally validated MCID exists for FCEAI, the observed day 28 separation (−2.5 points) represents a large effect size (Hedges g ≈ 1.33) and an approximate NNT of 3 to achieve ≥50% improvement, supporting clinical relevance. The treatment effect remained directionally consistent at day 56, with a between-group difference of −2.1 points (95% CI, −3.3 to −0.9; p = 0.004). Observed case visit counts were 17 versus 17 at day 28 and 16 versus 17 at day 56 for Immutalon versus placebo, respectively.

Responder rates (≥50% FCEAI reduction) on day 28 were higher with Immutalon (11/18, 61%) than placebo (5/18, 28%), giving an OR of 3.9 (95% CI, 1.1–14.1) and an absolute risk difference of +33% (95% CI, +2% to +64%). The NNT was approximately 3. Remission (FCEAI ≤3) occurred in 8/18 (44%) versus 3/18 (17%) at day 28 (OR 4.0; 95% CI, 0.85–18.9) and in 7/16 (44%) versus 3/17 (18%) at day 56 (OR 3.7; 95% CI, 0.78–18.1). Supporting MMRM analyses across days 7–28 were consistent with the primary inference (Table 2).

**Table 2:** Primary endpoint on day 28 and maintenance on day 56 (mITT; ANCOVA/MMRM with MI).

Outcome	Immutalon + SoC	Placebo + SoC	Between-group effect
Change in FCEAI to day 28 LS-mean (95% CI)	−4.8 (−5.6 to −4.0)	−2.3 (−3.1 to −1.5)	−2.5 (−3.7 to −1.3); p = 0.0007
Responders on day 28 (≥50%↓ FCEAI), n/n (%)	11/18 (61)	5/18 (28)	OR 3.9 (95% CI 1.1–14.1); RD +33% (95% CI +2% to +64%); p = 0.03
Remission on day 28 (FCEAI ≤3), n/n (%)	8/18 (44)	3/18 (17)	OR 4.0 (95% CI 0.85–18.9); p = 0.07
Change in FCEAI to day 56 (off-drug), LS-mean	−4.2	−2.1	−2.1 (95% CI −3.3 to −0.9); p = 0.004
Remission on day 56, n/n (%)	7/16 (44)	3/17 (18)	OR, 3.7 (95% CI, 0.78–18.1); p = 0.09

MMRM = Mixed model for repeated-measures, mITT = Modified intention-to-treat, SoC = Standard of care, CI = Confidence interval, OR = Odds ratio, FCEAI = Feline chronic enteropathy activity index, RD = Risk difference.

### Secondary clinical outcomes

Hierarchical testing of prespecified secondary endpoints confirmed broad clinical benefits (Table 3). Steroid-sparing effects were evident: 13/18 (72%) cats in the Immutalon arm remained steroid-free through day 28 compared with 7/18 (39%) in the placebo arm (OR 3.9; 95% CI, 1.1–14.1; absolute difference +33% [95% CI, +2% to +64%]; p = 0.04). The cumulative prednisolone dose to day 28 was lower with Immutalon (0 [0; 0.9] mg/kg) versus placebo (2.0 [0; 5.0] mg/kg), with a Hodges–Lehmann difference of −1.2 (95% CI, −2.3 to −0.1; p = 0.02).

**Table 3:** Secondary clinical outcomes on day 28 (mITT; hierarchy controlled where prespecified).

Outcome (Δ from baseline unless stated)	Immutalon + SoC (n = 18)	Placebo + SoC (n = 18)	Effect (95% CI)	p-value
Steroid-free status by day 28, n/n (%)	13/18 (72%)	7/18 (39%)	OR 3.9 (1.1–14.1); RD + 33% (+2% to +64%)	0.04
Cumulative prednisolone to day 28, mg/kg, median (interquartile range)	0 (0; 0.9)	2.0 (0; 5.0)	HL diff −1.2 (−2.3 to −0.1)	0.02
Vomiting episodes per 7 days	−2.8 ± 2.1	−1.1 ± 1.9	−1.7 (−3.1 to −0.4)	0.01
Stool score (0–3, higher = better)	+1.1 ± 0.9	+0.4 ± 0.8	+ 0.7 (+0.1 to +1.3)	0.02
Body weight (kg)	+0.22 ± 0.28	+0.07 ± 0.24	+ 0.15 (+0.01 to +0.30)	0.04
Owner-reported QoL (0–10, higher = better)	+1.3 ± 1.2	+0.4 ± 1.0	+ 0.9 (+0.3 to +1.5)	0.005
Ultrasound composite index (sum score)	−2.1 ± 1.8	−0.7 ± 1.6	−1.4 (−2.6 to −0.3)	0.02
Spec fPL, μg/L (whole cohort)	−1.2 ± 1.6	−0.2 ± 1.0	−1.0 (−1.9 to −0.1)	0.03

SoC = Standard of care, CI = Confidence interval, mITT = Modified intention-to-treat, OR = Odds ratio, QoL = Quality of life, Spec fPL = Specific feline pancreatic lipase, RD = Risk difference, HL = Hodges-Lehmann difference.

Additional day 28 clinical improvements included fewer vomiting episodes (−1.7/7 days; 95% CI, −3.1 to −0.4; p = 0.01), improved stool quality (+0.7 on a 0–3 scale; 95% CI, +0.1 to +1.3; p = 0.02), weight gain (+0.15 kg; 95% CI, +0.01 to +0.30; p = 0.04), and higher owner-reported quality of life (+0.9 on a 0–10 scale; 95% CI, +0.3 to +1.5; p = 0.005). Blinded ultrasound showed a greater improvement in the composite intestinal/pancreatic index (−1.4 points; 95% CI, −2.6 to −0.3; p = 0.02). Spec fPL declined more markedly with Immutalon (difference −1.0 µg/L; 95% CI, −1.9 to −0.1; p = 0.03).

### Analytical validation of EF-M2 clinical lots

All EF-M2 clinical lots met prespecified quality criteria, including an intact mass of 53.6 kDa; SDS-PAGE and RP-HPLC purity ≥96% and ≥97%, respectively; aggregates ≤0.5%; and endotoxin levels ≤0.05 EU/µg (measured 0.004–0.011 EU/µg). Biolayer interferometry confirmed micromolar-range binding to human and feline CLEC10A-Fc (K_D 0.85–1.1 µM) with GalNAc-dependent competition.

### PD biomarkers and clinicodynamic coupling

Prespecified PD markers shifted in the expected anti-inflammatory, M2-skewing direction. Relative to placebo, Immutalon increased the ARG1/iNOS ratio by +0.26 units (95% CI, +0.16 to +0.36; p < 0.001), increased IL-10 by +4.8 pg/mL (95% CI, +2.9 to +6.7; p < 0.001), and decreased TNF-α by −1.9 pg/mL (95% CI, −3.1 to −0.7; p = 0.01) on day 28. These biochemical changes correlated with clinical improvement: ΔARG1/iNOS versus ΔFCEAI (r = −0.57; 95% CI, −0.76 to −0.29; p = 0.001) and ΔIL-10 versus ΔFCEAI (r = −0.44; 95% CI, −0.68 to −0.12; p = 0.01). These findings establish a mechanistic link that supports the study’s biological rationale (Table 4).

**Table 4:** Pharmacodynamic biomarkers and clinicodynamic correlations.

Panel A. Change in PD biomarkers (mITT) from baseline to day 28				

Biomarker (Δ day 28−day 0)	Immutalon + SoC (n = 17 OC)	Placebo + SoC (n = 17 OC)	Between-group difference (95% CI)	p-value
ARG1/iNOS (a.u.)	+0.35 ± 0.17	+0.09 ± 0.12	+ 0.26 (+0.16 to +0.36)	<0.001
IL-10, pg/mL	+6.1 ± 3.2	+1.3 ± 2.6	+4.8 (+2.9 to +6.7)	<0.001
TNF-α, pg/mL	−2.4 ± 2.1	−0.5 ± 1.9	−1.9 (−3.1 to −0.7)	0.01

Panel B. Correlations between ΔFCEAI and changes in PD biomarkers on day 28 (mITT)				

**Prespecified correlation (Δ vs. ΔFCEAI; mITT)**	**r (95% CI)**		**p-value**	**n**

ΔARG1/iNOS as ΔFCEAI	−0.57 (−0.76 to −0.29)		0.001	34
ΔIL-10 as ΔFCEAI	−0.44 (−0.68 to −0.12)		0.01	34

SoC = Standard of care, CI = Confidence interval, PD = Pharmacodynamic, mITT = Modified intention-to-treat, IL-10 = Interleukin-10, TNF-α = Tumor necrosis factor-alpha, FCEAI = Feline chronic enteropathy activity index, ARG1/iNOS = Arginase-1 to inducible nitric oxide synthase ratio, a.u. = Arbitrary units

### Exploratory outcomes

In the exploratory microbiome subcohort (n = 15), 16S sequencing showed no significant within-arm shifts in Shannon diversity (EF-M2 Δ + 0.02 ± 0.21 vs. placebo Δ−0.03 ± 0.18; p = 0.42) or dominant taxa (Bacteroidetes: Firmicutes ratio Δ+0.03 vs. Δ + 0.01; p = 0.51). Fecal calprotectin decreased by −38 (−64; −11) µg/g with EF-M2 versus −12 (−33; +7) µg/g with placebo (between-group −26 µg/g; p = 0.08). Fecal α1-proteinase inhibitor decreased by −0.12 (−0.22; −0.02) mg/g versus −0.03 (−0.12; +0.06) mg/g (p = 0.09). Although not statistically conclusive, these findings suggest potential attenuation of mucosal inflammation without evidence of dysbiosis.

### Prespecified pancreatitis-positive subgroup

Within the pancreatitis-positive stratum (n = 20; 10/arm), baseline characteristics were comparable: Age 9.1 ± 3.2 versus 8.8 ± 3.1 years; male sex 6/10 versus 5/10; disease duration 7.0 (4–10) versus 6.5 (4–9) months; baseline FCEAI 7.6 ± 1.5 versus 7.5 ± 1.7 (all SMDs <0.20). Among these cats, Immutalon produced larger declines in Spec fPL (−2.1 ± 1.9 vs. −0.3 ± 1.1 µg/L; effect −1.8; p = 0.009) and greater improvement in the pancreatic ultrasound index (−1.2 ± 1.1 vs. −0.2 ± 0.9; effect −1.0; p = 0.01). Although interaction terms (p = 0.08–0.11) were suggestive but not conclusive, the concurrent improvement in intestinal and pancreatic indices supports a biologically coherent gut–pancreas axis effect (Table 5).

**Table 5:** Pancreatitis-positive subgroup (predefined; Spec fPL >5.3 μg/L and/or US features).

Outcome (Δ day 28−day 0)	Immutalon + SoC (n = 10)	Placebo + SoC (n = 10)	Effect (95% CI)	p-value	p-interaction (Tx × pancreatitis)
Spec fPL, μg/L	−2.1 ± 1.9	−0.3 ± 1.1	−1.8 (−3.1 to −0.5)	0.009	0.08
Pancreatic ultrasonography index (sum score)	−1.2 ± 1.1	−0.2 ± 0.9	−1.0 (−1.9 to −0.2)	0.01	0.11

SoC = Standard of care, CI = Confidence interval, Spec fPL = Specific feline pancreatic lipase

### Safety

Across ~304 total injections administered through day 56, TEAE rates were low and similar between groups (Table 6): 3/18 (17%) with Immutalon and 4/18 (22%) with placebo. Most events were Grade 1 (injection-site erythema/tenderness or transient lethargy). One Grade ≥2 event occurred in each group (transient ALT/AST increase ≈2.3× ULN with Immutalon™ that resolved without sequelae; unrelated intercurrent cystitis with placebo). There were no serious AEs, no deaths, and no discontinuations attributable to AEs (Fisher’s exact p = 0.99).

**Table 6:** Treatment-emergent adverse events (TEAEs), exposure, and relatedness (Security set).

Category (VCOG-CTCAE v2.1)	Immutalon + SoC (n = 18)	Placebo + SoC (n = 18)
Any TEAE, any grade, n (%)	3 (17)	4 (22)
Grade 1	3	4
Injection-site erythema/tenderness	2 (related)	3 (related)
Transient lethargy ≤24 h	1 (possibly related)	2 (possibly related)
Grade ≥2	1	1
ALT/AST increase (~2.3× ULN on day 21)	1 (possibly related); resolved without sequelae	0
Intercurrent cystitis	0	1 (unrelated)
Serious AEs (SAE)	0	0
Deaths	0	0
Discontinuation due to AE	0	0
Antidrug antibodies on day 28	0/17 tested (0%)	—

Injection-site reactions were Grade 1 with a median erythema diameter of 5 mm (Interquartile range (IQR) 3–7), resolving within 24 h. ADA were not detected (0/17 at day 28); later-time point ADA testing was not performed and is acknowledged as a limitation. Serial complete blood count and biochemical parameters showed no group-by-group interactions (all p > 0.20).

### Missing data and sensitivity analyses

The mITT analysis used multiple imputation under a missing at random assumption. Observed case counts at the primary endpoint (17 vs. 17 at day 28) were balanced. Supportive MMRM analyses across days 7–28 yielded conclusions consistent with the primary ANCOVA, and day 56 results demonstrated partial off-drug attenuation, retaining statistical significance.

Sensitivity analyses were concordant: Exclusion of two extreme improvers in the Immutalon arm (ΔFCEAI −9 and −10) and one paradoxical worsener on placebo (ΔFCEAI +4) yielded a day 28 between-group difference of −2.3 points (vs. −2.5 in the primary) with p = 0.0012. Winsorized ANCOVA and leave-one-out analyses produced similar inferences.

## DISCUSSION

### Overall clinical efficacy

In this multicenter, randomized, double-blind, placebo-controlled add-on trial in client-owned cats with CE (with and without chronic pancreatitis), EF-M2 (Immutalon) produced clinically and statistically significant reductions in disease activity, with partial persistence after treatment cessation. The primary endpoint, change in FCEAI from baseline to day 28, favored EF-M2 over placebo by a least-squares mean difference of −2.5 points (95% CI −3.7 to −1.3; p = 0.0007). Responder rates (≥50% FCEAI reduction) were 61% in the EF-M2 arm versus 28% in the placebo arm, yielding an approximate NNT of ≈3 for this stringent criterion. Importantly, a clinically coherent pattern extended to day 56 (off-drug), where the EF-M2 cohort maintained a larger cumulative improvement in FCEAI (Δ = −4.2) than placebo (Δ = −2.1), with a between-group difference of −2.1 (p = 0.004). In the absence of an external MCID for FCEAI, the combination of a −2.5-point separation, Hedges g ≈ 1.33, and NNT ≈ 3 provides actionable anchors for clinical interpretation under standardized diet/B12 care. These effects were accompanied by steroid-sparing (72% steroid-free vs. 39% on placebo by day 28), lower cumulative prednisolone dose, and favorable changes in owner-reported symptoms (vomiting, stool quality) and body weight. Together, the data indicate that a short-course of EF-M2 can shift the clinical trajectory beyond the treatment window under a standardized SoC algorithm [20].

The magnitude of FCEAI improvement relative to placebo at day 28 is not only statistically robust but also pragmatically meaningful. A least-squares mean separation of −2.5 points with an NNT of ~3 compares favorably with effect sizes typically considered actionable in companion-animal gastroenterology, where background care absorbs much of the modifiable variance [20]. The steroid-sparing effect is particularly important, given the long-recognized trade-off between rapid symptom control and cumulative glucocorticoid toxicity [4]. More cats avoided prednisolone entirely by day 28, and cumulative exposure was lower in the EF-M2 arm while achieving superior symptom control. That benefits persisted to day 56 off-drug suggests a disease-modifying influence on myeloid tone in the gut–pancreas axis, pending confirmation in longer studies [1, 21, 22].

### Mechanistic basis and PD coherence

EF-M2 is an analytically defined, α-GalNAc–presenting variant of the Vitamin-D–binding-protein platform designed to target the myeloid lectin CLEC10A and bias macrophages toward an IL-10/ARG1-dominant (“M2-leaning”) phenotype under tightly controlled endotoxin limits [10, 23–26]. In the present trial, serum PD markers moved in step with clinical change: ARG1/iNOS increased, IL-10 increased, and TNF-α declined, with dose-consistent directionality and clinicodynamic correlations (ΔARG1/iNOS vs ΔFCEAI r ≈ −0.57). This coherence strengthens the causal interpretation that EF-M2’s intended macrophage-programming contributes to symptom relief.

Mechanistically, the precision glycomodulation underlying EF-M2, defined monosaccharide identity, extremely low endotoxin, and receptor-engaging α-GalNAc display, was purpose-built to avoid the heterogeneity, contaminants, and cytokine artifacts that complicated the interpretation of early serum-derived “GcMAF 1.0.” CLEC10A functions as a contextual “dimmer switch” in Th2-permissive tissue niches, where α-GalNAc ligation amplifies pro-resolution outputs without triggering broad Toll-like receptor (TLR)-type inflammatory activation [10, 23–27]. The trial’s PD signature and durable off-drug benefit align with an M2-biased reframing of the intestinal myeloid compartment [21].

### Cross-species evidence and translational plausibility

Controlled, blinded evidence targeting macrophage state in feline enteropathy has historically been sparse [28]. Cross-species data from a randomized, double-blind, placebo-controlled canine osteoarthritis trial using the same EF-M2 ligand demonstrated dose-frequency-dependent improvements in pain and function, accompanied by increased ARG1/iNOS and IL-10 and decreased TNF-α, with biomarker changes correlating with clinical benefit, precisely the PD, clinical alignment observed in this feline trial [29]. This canine dataset provides external plausibility for EF-M2 as a mechanistically anchored adjunct capable of modifying myeloid programs across tissues and species. To the best of our knowledge, the present study is the first randomized, double-blind evaluation of an analytically defined, M2-biased macrophage ligand as an add-on therapy in feline enteropathy, and its convergence with the canine dataset suggests a conserved, CLEC10A-centered biology worth systematic development [2, 3, 22, 28].

Although the target tissues differ, synovium in canine osteoarthritis versus the gut–pancreas axis in feline CE, the shared presence of iNOS^+^/TNF-α^+^ macrophages driving pathology provides a unifying mechanistic substrate. The concordant increases in IL-10 and ARG1/iNOS, decreases in TNF-α, and predictive value of ΔARG1/iNOS for clinical response in both species support the hypothesis that α-GalNAc–CLEC10A engagement rebalances myeloid tone toward resolution rather than broad immunosuppression [2, 3, 22, 28, 29].

### Triaditis-axis and pancreatic outcomes

Prespecified analyses along the triaditis (gastro-pancreatic) axis revealed greater benefit in cats with pancreatitis features [30]. In this subgroup, the mean change in Spec fPL was −2.1 µg/L with EF-M2 versus −0.3 µg/L with placebo (p = 0.009), and the pancreatic ultrasound index improved more markedly with EF-M2. Concurrent improvement across intestinal symptoms (FCEAI, stool, vomiting, weight), pancreatic biomarkers, and PD markers suggests that macrophage reprogramming in ductal/peripancreatic niches contributes to disease quieting [30–32]. Although imaging was pragmatic and subgroup analyses exploratory, the alignment across clinical, organ-axis, and PD domains strongly argues against a purely symptomatic mechanism [10, 22, 23].

The PD suite (ARG1/iNOS, IL-10, TNF-α) shifted coherently at day 28 with p ≤ 0.01, and the magnitude of ARG1/iNOS elevation predicted improvement in FCEAI. This chain, CLEC10A engagement → IL-10 upregulation → ARG1 activation → tissue repair and reduced TNF-α, provides a plausible pathway linking receptor activation to clinical benefit [33-34].

### Safety and tolerability

EF-M2 achieved clinically significant improvement without evidence of broad immunosuppression, steroid-related toxicities, or gut-flora disruption. More cats were steroid-free with EF-M2, and cumulative prednisolone exposure was lower. TEAEs were infrequent, mild, and comparable to placebo, with no serious AEs. Serial hematology and chemistry profiles did not reveal cytopenias or infection-like signals. Stool quality improved rather than deteriorated and exploratory 16S profiling showed no significant changes in Shannon diversity or dominant taxa. These observations support immune rebalancing rather than global suppression.

### Comparative mechanistic distinction from conventional immunosuppressants

Mechanistically, EF-M2 engages CLEC10A and biases macrophages toward IL-10/ARG1-dominant states, a targeted approach distinct from glucocorticoids and calcineurin inhibitors. Glucocorticoids broadly repress transcriptional networks (NF-κB/AP-1) and carry risks such as diabetes and muscle wasting, while cyclosporine suppresses T-cell activation with associated infection risk. EF-M2 aims to reset myeloid tone without ablating antimicrobial competence, providing steroid-sparing control with placebo-like safety. Cross-species evidence from the canine RCT reinforces this favorable risk–benefit profile.

### Integration of clinical, organ-axis, and mechanistic layers

This trial integrates multiple levels of evidence: (i) Clinical indices (FCEAI, diaries, weight, responder status), (ii) organ-specific markers (Spec fPL, blinded intestinal/pancreatic ultrasonography), and (iii) macrophage-linked PD markers (ARG1/iNOS, IL-10, TNF-α).

The directional concordance, dose-consistent time course, and clinicobio marker correlation collectively satisfy multiple plausibility criteria connecting intended CLEC10A-centered macrophage-programming to observed clinical benefit. This is the first feline enteropathy RCT to prospectively link macrophage-specific biomarkers with clinical outcomes using an analytically standardized CLEC10A ligand [1, 2, 4, 10, 23, 32, 35–39].

## CONCLUSION

This randomized, double-blind, placebo-controlled add-on trial demonstrates that EF-M2 (Immutalon), an analytically defined CLEC10A-targeted macrophage-programming agent, provides clinically meaningful benefits for cats with CE, including those with concurrent chronic pancreatitis. EF-M2 produced significantly greater reductions in FCEAI at day 28 compared with placebo (least-squares mean difference −2.5; p = 0.0007), with 61% of treated cats achieving ≥50% improvement versus 28% on placebo (NNT ≈ 3). Improvements in vomiting, stool quality, body weight, and quality of life, along with a more pronounced decline in Spec fPL and enhanced intestinal/pancreatic ultrasonographic indices, underscore the multidimensional therapeutic effect. Importantly, partial persistence of clinical benefit to day 56 suggests a biology-modifying mechanism rather than short-lived symptomatic relief. PD changes, higher ARG1/iNOS ratio, increased IL-10, and reduced TNF-α, correlated with clinical improvement, supporting a coherent mechanistic link between CLEC10A-mediated myeloid reprogram-mming and disease control.

Key strengths include the randomized, double-blind, placebo-controlled design; rigorous masking procedures; a standardized SoC backbone (elimination diet, B12 normalization, and rescue algorithm); triangulated endpoints integrating clinical activity, pancreatic markers, and macrophage-linked PD signals; inclusion of an off-drug assessment demonstrating persistence of benefit; and the use of an analytically defined ligand with validated receptor binding, glycoidentity, and endotoxin control.

Important limitations include the modest sample size; the short treatment and observation window (4-week dosing; 8-week total); absence of post-treatment biopsies; pragmatic, site-level ultrasound without central adjudication; lack of tissue-level PD confirmation; absence of PK/PD modeling; exploratory and underpowered microbiome and fecal biomarker data; incomplete long-term safety characterization; and the absence of an externally validated MCID for FCEAI. Future development should incorporate multiple EF-M2 lots, orthogonal potency assays, and extended safety follow-up to improve generalizability and mechanistic resolution.

These findings support myeloid programming as a tractable adjunctive strategy in feline CE and triaditis, offering measurable symptom relief, steroid-sparing benefit, and mechanistic coherence with macrophage-linked PD markers. Cross-species consonance with a blinded canine RCT evaluating the same ligand suggests a conserved CLEC10A-centered biology relevant to inflammatory diseases dominated by myeloid dysregulation. Short-course EF-M2 appears capable of resetting the inflammatory milieu in ways that partially endure beyond active dosing, supporting its potential integration into tiered SoC pathways aimed at reducing glucocorticoid dependence.

Next-step trials should include 6–12-month multi center studies powered for durability, steroid-sparing outcomes, and triaditis-axis effects; incorporate PK/PD mapping and receptor-engagement assays; embed tissue-level biomarker phenotyping; and employ quantitative ultrasound with central review. If replicated under larger, blinded conditions with lot-traceable analytical controls, EF-M2 could advance as a biology-modifying adjunct that addresses underlying inflammatory mechanisms rather than providing symptomatic suppression alone.

Taken together, the clinical, organ-axis, and mechanistic coherence demonstrated in this trial positions EF-M2 as a promising, well-tolerated, receptor-guided immunobiologic with the potential to transform adjunctive management strategies for feline CE and triaditis. Continued development in larger and longer studies is warranted to confirm durability, refine patient selection, and firmly establish EF-M2 as a novel therapeutic option in veterinary gastroenterology.

## DATA AVAILABILITY

The minimal dataset for FELINE-TRIAD-1, comprising anonymized individual-level values for FCEAI components (vomiting frequency, stool quality, appetite, weight, and quality of life), cumulative steroid exposure, Spec fPL, ultrasonography indices, and serum biomarkers (ARG1/iNOS, IL-10, TNF-α), together with a data dictionary and reproduction-check outputs, is provided as Supplementary Material in a single Excel workbook (three sheets: Dataset, data_dictionary, and reproduction_check) published with this article. All data are anonymized and comply with institutional and national regulations.

## AUTHORS’ CONTRIBUTIONS

EP and RM: Conceptualization. EP and MJ: Methodology. NR, AS, and CG: Clinical investigation. DK, RM, JS, and CG: Formal analysis. NR, AS, and DK: Resources and data curation. EP and CG: Writing—original draft. RM, DK, and MJ: Writing—review and editing. MJ: Visualization. EP: Supervision and project administration, and funding acquisition. All authors have read and approved the published version of the manuscript.
